# Orthostatic Intolerance and Postural Orthostatic Tachycardia Syndrome in Joint Hypermobility Syndrome/Ehlers-Danlos Syndrome, Hypermobility Type: Neurovegetative Dysregulation or Autonomic Failure?

**DOI:** 10.1155/2017/9161865

**Published:** 2017-02-12

**Authors:** Claudia Celletti, Filippo Camerota, Marco Castori, Federica Censi, Laura Gioffrè, Giovanni Calcagnini, Stefano Strano

**Affiliations:** ^1^Physical Medicine and Rehabilitation, Umberto I Hospital, Rome, Italy; ^2^Unit of Clinical Genetics, San Camillo-Forlanini Hospital, Rome, Italy; ^3^Department of Cardiovascular, Dysmetabolic and Aging-Associated Diseases, Italian Institute of Health, Rome, Italy; ^4^Department of Heart and Great Vessels “A. Reale”, Sapienza University of Rome, Rome, Italy

## Abstract

*Background*. Joint hypermobility syndrome/Ehlers-Danlos syndrome, hypermobility type (JHS/EDS-HT), is a hereditary connective tissue disorder mainly characterized by generalized joint hypermobility, skin texture abnormalities, and visceral and vascular dysfunctions, also comprising symptoms of autonomic dysfunction. This study aims to further evaluate cardiovascular autonomic involvement in JHS/EDS-HT by a battery of functional tests.* Methods*. The response to cardiovascular reflex tests comprising deep breathing, Valsalva maneuver, 30/15 ratio, handgrip test, and head-up tilt test was studied in 35 JHS/EDS-HT adults. Heart rate and blood pressure variability was also investigated by spectral analysis in comparison to age and sex healthy matched group.* Results*. Valsalva ratio was normal in all patients, but 37.2% of them were not able to finish the test. At tilt, 48.6% patients showed postural orthostatic tachycardia, 31.4% orthostatic intolerance, 20% normal results. Only one patient had orthostatic hypotension. Spectral analysis showed significant higher baroreflex sensitivity values at rest compared to controls.* Conclusions.* This study confirms the abnormal cardiovascular autonomic profile in adults with JHS/EDS-HT and found the higher baroreflex sensitivity as a potential disease marker and clue for future research.

## 1. Introduction

Ehlers-Danlos syndrome (EDS) is an umbrella term for a group of heritable soft connective tissue disorders mainly characterized by generalized joint hypermobility, skin texture abnormalities, and visceral and vascular fragility or dysfunctions [1]. Current nosology identifies six major EDS variants, with the classical, hypermobility, and vascular types being the most common [1]. More recently, different studies suggested and partly demonstrated a clinical overlap between EDS, hypermobility type (EDS-HT), and the joint hypermobility syndrome (JHS) [2, 3]. JHS is to date defined as an underdiagnosed rheumatologic condition showing generalized joint hypermobility, chronic musculoskeletal pain, and additional findings and presenting a common familial aggregation [4]. The diagnosis of EDS is confirmed by molecular tools in most types except for EDS-HT and the cognate JHS, both remaining clinical diagnoses based on available criteria [4, 5]. At the moment, the prevalent opinion is to provisionally consider JHS and EDS-HT a single entity (i.e., JHS/EDS-HT) [2]. In JHS/EDS-HT, generalized joint hypermobility with recurrent joint dislocations and chronic moderate to severe pain were the most frequent and severe complaints, but also muscle cramps, tendinitis, headache, and fatigue are frequently reported [6]. Impaired proprioception, postural control, and muscular strength are factors that may contribute to joint instability [7]. The clinical spectrum associated with JHS/EDS-HT is not limited to the musculoskeletal apparatus. Among the common visceral manifestations of JHS/EDS-HT, symptoms possibly linked to an underlying autonomic dysfunction were repeatedly reported [8, 9]. In particular, different authors described orthostatic intolerance (OI) and postural orthostatic tachycardia symptoms (POTS) in JHS/EDS-HT [10–13].

POTS is one of the most common manifestations of OI and is defined as a sustained heart rate (HR) increase of ≥30 bpm or increase of HR to ≥120 bpm usually within the first 10 minutes of orthostasis associated with symptoms of OI but without orthostatic hypotension (OH), which is in turn defined as a drop of >20 mmHg in systolic blood pressure (BP) or >10 mmHg in diastolic BP independently of changes in HR [14, 15]. The diagnosis of POTS is instrumental and the association with symptoms is not linear. Therefore, there are patients manifesting complaints likely related to some autonomic dysfunction but not meeting the diagnostic criteria of POTS. The term OI is used to describe patients developing symptoms on standing or head-up tilt (HUT) but not fulfilling the criteria of POTS [16].

This study aims to evaluate the autonomic response profile and related symptoms in JHS/EDS-HT by HUT-test and spectral analysis of heart rate and blood pressure variability.

## 2. Materials and Methods

### 2.1. Subjects

Patients were selected from those attending the Joint Hypermobility Outpatient Service at the Umberto I University Hospital in Rome. The diagnosis of JHS and EDS-HT was established by applying published diagnostic criteria. In particular, generalized joint hypermobility was assessed by the Beighton score (BS) with a maximum score of nine and a cut-off of four (≥4/9) for JHS and five (≥5/9) for EDS-HT [17]. Then, Villefranche criteria were used for EDS-HT [1], whereas Brighton score was applied for JHS [4]. Although a consensus is still lacking on the correct procedure of performing a satisfactory differential diagnosis, partially overlapping disorders were excluded on clinical and, if needed, molecular grounds as detailed elsewhere [18]. None of the patients had diabetes, heart failure, severe arterial hypertension, or cardiac arrhythmias, nor had any medications that might influence autonomic cardiovascular regulation. All the participants gave their informed consent before enrolment.

### 2.2. Procedure

All patients were studied at the Autonomic Cardiovascular Laboratory and Syncope Unit of the Department of Heart and Great Vessels “A. Reale”, Umberto I University Hospital in Rome. To exclude potentially preexisting cardiovascular disease not linked to the underlying genetic disorder, all patients underwent a full cardiovascular evaluation comprising complete history and physical examination, 12-lead electrocardiogram (ECG), 24 H Holter, hematocrit, and echocardiogram. Selected patients underwent cardiovascular reflex tests comprising deep breathing, Valsalva maneuver, 30/15 ratio, handgrip test, and HUT.

During the tests, continuous arterial BP was noninvasively recorded using a photo-plethysmographic technique (CNSystems Task-Force Monitor 3040i). The HR and BP beat-to-beat variability series were calculated according to the HR variability (HRV) guidelines [19]. Each beat was obtained by numerical recognition of R waves (R-R, tachogram). The systolic values were detected in the same cardiac cycle (S-S, systogram).

### 2.3. Cardiovascular Reflex Tests

#### 2.3.1. Deep Breathing

Participants breathed maximally at a frequency of 6 breaths per minute, following the lead of an oscillating ball on a computer screen. A total of 8 breathing cycles were recorded and the test was repeated after 2 min of rest. The deep breathing test was considered normal if heart rate variation was 15 beats/min or more, borderline if 11–14 beats/min, and pathological if 10 beats/min or less [20]. The HR range was calculated as a measure of parasympathetic reactivity [21].

#### 2.3.2. Valsalva Maneuver

Participant blew into a mouthpiece between 40 and 50 mmHg for 15 sec [21]. The maneuver was repeated 3 times or until at least 2 reproducible BP recordings were obtained. Faulty trials (inadequate pressure or duration) were excluded. In between trials, 3 min of rest was provided to stabilize HR and BP. The best-performed maneuver was selected for evaluation. The Valsalva ratio (VR) was calculated (parasympathetic measure), and the 4 phases in the BP response were quantified (sympathetic reactivity) [21]. Respect to baseline, the maximal drop of systolic and diastolic BP (SBP and DBP) at phase II early (IIE), SBP and DBP at phase II late (IIL), and overshoot at phase IV during Valsalva maneuver were computed. Difference between SBP and DBP from phase IIE to phase IIL and overshoot at phase IV during the maneuver were considered indexes of sympathetic vascular peripheral response.

#### 2.3.3. Early Heart Rate Response to Standing Up (30-15 Ratio)

Subjects were asked to stand at rest in the supine position for 5 min and then to assume the standing position without help as quickly as possible according to his/her physical abilities. The cardiac chronotropic response characteristic is expressed by the ratio 30 : 15, that is, the ratio between the longest R-R interval after the maneuver (around the thirtieth beat) and the shortest R-R interval around the fifteenth beat. Results were evaluated considering as normal a ratio ≥1.04, as borderline when the ratio value was between 1.01 and 1.03, and as pathological when the value is ≤1.00 [20].

#### 2.3.4. BP Response to Sustained Handgrip

Handgrip was maintained at 30 percent of maximal voluntary contraction until the maximum time of 5 minutes, using a dynamometer. Pressure was measured every minute until the end of the test. Magnitude of the response was given by the difference between the diastolic BP measured just before the release of the dynamometer and that measured before the test. Handgrip test was considered successfully performed if patients were able to maintain a constant effort for at least 1 minute.

#### 2.3.5. HUT

Participants rested quietly for 5 minutes. Baseline HR and BP were calculated as the mean from 40 seconds to 10 seconds before tilting. Then, the table was slowly tilted upright to an angle of 70° for a maximum of 20 minutes. Patients were asked to report all symptoms and the test was aborted when either orthostatic symptoms or pain became intolerable. OH was defined as a sustained diastolic BP drop of at least 10 mmHg or a systolic BP drop of at least 20 mmHg for normotensive subjects [22]. Vasovagal syncope was defined as a sudden BP and HR decline coupled with loss of consciousness. POTS was defined as a sustained HR rise of at least 30 bpm or a HR of at least 120 bpm in the first 10 min of tilt, without concomitant OH [23]. OI was the term used to define symptoms at HUT in the absence of the abovementioned criteria.

#### 2.3.6. Spectral Analysis of Cardiovascular Variability Signals

Cardiovascular autonomic function was assessed by means of short-term power spectral analysis of HR variability, arterial BP variability, and spectral estimation of baroreflex sensitivity (*α*-index) at rest and during tilt. Short-term power spectra of HR variability signals were estimated by autoregressive modeling over 250 consecutive beats. According to the HRV guidelines [19], the powers of the two main frequency bands of R-R variability were considered: high-frequency (HF) component (ranging between 0.15 and 0.40 Hz, indirectly reflecting efferent vagal activity and synchronized with the respiratory frequency) and low-frequency (LF) component (ranging between 0.04 and 0.15 Hz which reflects sympathetic and parasympathetic control and always increases during sympathetic activation) [24]. LF and HF powers were computed in absolute units (ms^2^) and transformed into normalized units (n.u.) (i.e., the percentage of the total spectral power minus the very-low-frequency component) in order to minimize the influence of changes in total power on the LF and HF power values. We also computed the LF to HF ratio (LF/HF), which is considered to be an index of sympathovagal balance.

#### 2.3.7. Baroreflex Sensitivity Estimation (*α*-Index)

Baroreflex sensitivity (*α*-index) [25] was computed as the ratio between the spectral power of tachogram and systogram, in the HF (*α*_HF_) and LF (*α*_LF_) bands, respectively, and then summed (*α*_LF+HF_). *α*-index was expressed as ms/mmHg.

Results were compared with those obtained in a group of 23 age- and sex-matched healthy subjects who underwent the same HUT-test protocol.

### 2.4. Statistical Analysis

Student's paired *t*-test was used to compare differences in the spectral variables investigated between rest and tilt. Student's unpaired *t*-test was used to compare differences in the spectral variables between groups. A *P* value of <0.01 was considered to indicate statistical significance.

## 3. Results

Thirty-five patients (6 men and 29 women, mean age of 35 ± 14 years) with JHS/EDS-HT were included.

### 3.1. Cardiovascular Reflex Tests

A summary of the cardiovascular reflex tests results was reported in Table 1.

Deep breathing test and 30/15 ratio gave normal results in all patients. Valsalva ratio was also normal in all patients who finished the test (54.3%), while 31.4% patients were not able to conclude the test due to breathing difficulties and incoordination. All patients failed to complete the sustained handgrip test, because pain and fatigue occurred in the early stage of the text (<1 min), and this did not allow the collection of valid BP values.

Among the 22 patients that completed the Valsalva maneuver, the increase of BP between early phase II and late phase II and the overshoot in late phase IV were observed (Table 2).

By comparing the Valsalva maneuver between JHS/EDS-HT patients with POTS (see below) and those without the criteria for POTS and OH (see below), no significant differences were observed (Table 3).

### 3.2. HUT-Test and Spectral Analysis

The HR and BP responses after HUT are showed in Table 4.

Postural tachycardia was common in JHS/EDS-HT. Seventeen (48.6%) met the criteria of POTS, 11 (31.4%) had OI, while 7 (20%) gave normal results. Only one (3%) had OH while a normal pressure response was observed in the other 34 (97%).

Results of spectral analysis were summarized in Table 5. Analysis of HR showed comparable results between JHS/EDS-HT and controls. At rest, JHS/EDS-HT patients showed significant higher BRS values compared to controls, while these values became not statistically significant at tilt. BRS values at rest showed further differences by separating the patients' sample in two subgroups (i.e., POTS and non-POTS patients). In particular, we observed a significant higher BRS in the JHS/EDS-HT group with POTS versus controls (*α*LF + HF = 68.4 ± 41.3 ms/mmHg in POTS versus 35.5 ± 11.0 ms/mmHg in controls, *P* < 0.005), while non-POTS patients showed a slight but not statistically significant increase of the BRS compared to controls (*α*LF + HF = 49.2 ± 29.6 ms/mmHg versus 35.5 ± 11.0 ms/mmHg in controls).

## 4. Discussion

This work confirmed a common perturbation of autonomic regulation of the cardiovascular function in adults with JHS/EDS-HT. POTS and OI were the more commonly observed profiles at HUT, while OH, as a genuine form of autonomic failure, was rare in our sample. Interestingly, we observed a difficulty in completing both the handgrip test and the Valsalva maneuver among the JHS/EDS-HT patients. It is possible that the hand problems related to fatigue and joint pain or instability (so commonly observed in JHS/EDS-HT [26]), as well as the lack of proprioception [27], might have contributed to this pathological outcome. During the Valsalva maneuver, we registered an increase of BP between early phase II and late phase II, and the overshoot in late phase IV in JHS/EDS-HT, as expected in physiological peripheral sympathetic vasoconstriction. The recent finding of small fiber neuropathy as a common finding in EDS, also comprising JHS/EDS-HT, is an intriguing conundrum in light of our findings [28]. In fact, the presence of small fiber neuropathy could identify a clinical subgroup of POTS (a.k.a. “neuropathic” POTS) [29]. In this category of POTS the phase IV overshoot of the Valsalva maneuver is lower than controls, a finding that we were not able to replicate in our sample. This implies a much complex pathogenesis for dysautonomic symptoms in this disorder.

Considering the results obtained with the HUT-test, we found POTS in ~50% JHS/EDS-HT patients and a high rate of OI in accordance with previous studies [10]. POTS and OI have been frequently described in chronic fatigue syndrome and fibromyalgia, a fact that suggests that these conditions may share deconditioning. Deconditioning could then operate in concert with “somatic hypervigilance” to lead to a disturbing mismatch between physiological responses and perception in some individuals [30]. This could then lead to a vicious cycle of less activity and more deconditioning often worsened by excessive “medicalization.” Accordingly, it seems reasonable that exercise-based rehabilitation may be effective in the medium- and long-term treatment of POTS and related conditions with deconditioning [30]. In fact, it has been demonstrated that, during acute exercise, POTS patients have excessive increases in BP and reduced stroke volume at absolute workload compared to sedentary controls without intrinsic abnormality of HR regulation. In addition, cardiac remodeling and blood volume expansion associated with exercise training increase physical fitness and improve performance in these patients [31]

Assessment of autonomic nervous system activity by power spectral analysis of HRV and BPV has attracted growing interest and is already consolidated over the years. Spectral analysis is a noninvasive tool for evaluating the neural mechanisms controlling HR and BP [19]. It identifies the harmonic components of a signal and shows how its power is distributed as a function of frequency. Spectral analysis of HR did not show significant differences in the response to the tilt maneuver between JHS/EDS-HT and controls. In response to orthostatic stimulation, we observed comparable changes in the HR markers of sympathetic activation and parasympathetic withdrawal in both groups. An interesting finding of our study is that the BRS of JHS/EDS-HT patients, at rest, is significantly higher than controls and this difference is more marked in the subgroup of patients with POTS. Different hypothesis can explain the BRS variations. The resting HR seems to be influenced by exercise. Different studies have demonstrated reduction of intrinsic HR induced by long-term competitive training in athletes compared with sedentary individuals [32, 33]. Another possible mechanism to explain the abnormalities of BRS in JHS/EDS-HT may be linked to the reduction of artery stiffness in the elderly [34]. Since the deformation of the baroreceptors rather than direct intravessel pressure during acute changes in arterial pressure is required to initiate neural firing, stiffness of the large elastic arteries with baroreceptors (i.e., carotid artery, the aortic arch) could be associated with a decrease in BRS [34]. From this perspective, it should be possible to speculate that the modification of the connective tissue which characterizes JHS/EDS-HT may modify vessels compliance and should explain the differences observed in the baroreflex function.

### 4.1. Limitations

This study have some limitations: firstly autonomic cardiovascular symptoms are not tested using specific questionnaire (e.g., the Composite Autonomic Scoring Scale) and no correlation between clinical and instrumental results is present; the number of patients is reduced; symptoms and tests have not been reevaluated during the natural history of the JHS/EDS-HT in order to analyze possible variations or the response to clinical and pharmacological therapies. Future studies are needed in order to study the changes of autonomic regulation before and after therapeutic exercise or after low intensity sports activities and they might be also introduced in future research with the aim of identifying more reliable markers for the effects rehabilitation in such a complex disorder. Symptoms should be observed in a longer period following the different phases of the syndrome; moreover it should be useful to better investigate the vascular stiffness in JHS/EDS-HT patients and its possible role in the genesis of the results founded.

## Figures and Tables

**Table 1:** Response to cardiovascular reflex tests.

Test	Normal number (%)	Borderline number (%)	Pathological number (%)	Not evaluable number (%)
Deep breathing	35/35 (100)	—	—	—
Valsalva ratio	19/35 (54.3)	3/35 (8.5)	—	13/35 (31.4)
30/15 ratio	17/35 (48.57)	18/35 (51.4)	—	
Sustained handgrip	—	—	—	35/35 (100)

**Table 2:** Valsalva maneuver in JHS/EDS-HT patients who performed the test (#22).

Variable	mmHg ± DS
Baseline SBP/DBP	116.7/73.9 ± 15.3/11.2
Maximal drop of SBP during early phase II	23.5 ± 9.96
Maximal drop of DBP during early phase II	4.8 ± 4.8
SBP at late phase II	125.9 ± 17.3
DBP at late phase II	85.4 ± 12.2
SBP at late phase IV	137.6 ± 21.2
DBP at late phase IV	84.0 ± 14.8
Difference between late phase II SBP and early phase II SBP	32.7 ± 15.6
Difference between late phase II DBP and early phase II DBP	17.6 ± 6.8

*Normal values*: Maximal drop of mean blood pressure (MBP) during early phase II: ≥20 mmHg; systolic blood pressure (SBP) and diastolic blood pressure (DBP) at late phase II: ≥baseline; SBP and DBP at late phase IV: ≥baseline.

**Table 3:** Valsalva maneuver: comparison of JHS/EDS-HT patients stratified according to the heart rate response to tilting (#21).

POTS (10/35)		Non-POTS (11/35)		*P*
Variable	mmHg ± ds	Variable	mmHg ± ds	
Baseline SBP/DBP	117/74.5 ± 11.6/6.8	Baseline SBP/DBP	111/70 ± 9.2/8	*P* = 0.23
Maximal drop of SBP during early phase II	24.5 ± 10.6	Maximal drop of SBP during early phase II	21.9 ± 24.3	*P* = 0.7
maximal drop of DBP during early phase II	3.9 ± 9.1	Maximal drop of DBP during early phase II	3.6 ± 16	*P* = 0.7
SBP at late phase II	125 ± 13.1	SBP at late phase II	118 ± 18	*P* = 0.48
DBP at late phase II	89.5 ± 11.6	DBP at late phase II	84 ± 17	*P* = 0.4
SBP at late phase IV	142.5 ± 19.6	SBP at late phase IV	132 ± 22.4	*P* = 0.27
DBP at late phase IV	87 ± 15.6	DBP at late phase IV	78.6 ± 15.1	*P* = 0.22
Difference between late phase II SBP and early phase II SBP	31.8 ± 17.9	Difference between late phase II SBP and early phase II SBP	30.8 ± 9.2	*P* = 0.6
Difference between late phase II DBP and early phase II DBP	18.9 ± 9	Difference between late phase II DBP and early phase II DBP	18. 8 ± 6.3	*P* = 0.7

Normal values: Maximal drop of mean blood pressure (MBP) during early phase II: ≥20 mmHg; systolic blood pressure (SBP) and diastolic blood pressure (DBP) at late phase II: ≥baseline; SBP and DBP at late phase IV: ≥baseline.

**Table 4:** Heart rate and blood pressure responses after head-up tilt test.

	HR response ratio (%)	HR mean difference (bpm ± SD)		BP response ratio (%)	SBP mean difference (mmHg ± DS)	DBP mean difference (mmHg ± DS)
*Normal* (HR increase between 10 and 19 bpm)	7/35 (20)	12.5 ± 2.3	*Normal*	34/35 (97)	5.9 ± 5.8	6.5 ± 5.8
*POTS* (HR increase > 30 bpm)	17/35 (48.6)	32 ± 6.5	*Orthostatic hypotension* (reduced peripheral vessel response)	1/35 (3)	−25	−15
*Reduced orthostatism tolerance* (HR increase from 20 to 29 bpm)	11/35 (31.4)	20.4 ± 1.4				

HR: heart rate. BP: blood pressure. DBP: diastolic blood pressure. SBP: systolic blood pressure.

**Table 5:** Spectral parameters of heart rate variability and baroreflex sensitivity estimation.

	JHS/EDS-HT (*N* = 35)			Controls (*N* = 23)		
	Rest	TILT	*P*	Rest	TILT	*P*
			(rest versus tilt)			(rest versus tilt)
Mean RR [s]	0.91 ± 0.14	0.73 ± 0.12	<0.001	0.89 ± 0.10	0.76 ± 0.1	<0.001
Tot power [msec^2^]	3281 ± 2875	2187 ± 1423	n.s	2419 ± 1430	1961 ± 957	n.s
LF power (u.n.)	57.0 ± 21.7	82.7 ± 10.9	<0.001	57.5 ± 18.4	82.3 ± 14.6	<0.001
HF power (u.n.)	43.0 ± 21.7	17.3 ± 10.9	<0.001	42.5 ± 18.4	17.7 ± 14.6	<0.001
LF/HF	2.18 ± 1.99	8.36 ± 8.75	<0.001	1.98 ± 1.96	12.0 ± 13.0	<0.002
*α*_(LF+HF)_ [msec/mmHg]	59.1 ± 36.9^**++**^	22.0 ± 9.6	<0.001	35.5 ± 11.0	18.5 ± 8.6	<0.001
*α*_LF_ [msec/mmHg]	21.6 ± 11.8^**+**^	11.5 ± 5.8	<0.001	15.9 ± 5.8	10.8 ± 4.3	<0.001
*α*_HF_ [msec/mmHg]	37.5 ± 29.4^**++**^	10.4 ± 5.2	<0.001	19.7 ± 7.8	7.7 ± 4.9	<0.005

LF: low frequency

HF: high frequency

*α*: baroreflex sensitivity

^+^ *P* < 0.02 JHS/EDS-HT versus controls; ^++^*P* < 0.001 JHS/EDS-HT versus controls.

## Abbreviations

BP: Blood pressure
bpm: Beats per minute
BRS: Baroreflex sensitivity
DBP: Diastolic blood pressure
ECG: Electrocardiogram
EDS: Ehlers-Danlos syndrome
EDS-HT: Ehlers-Danlos syndrome, hypermobility type
HF: High-frequency
HR: Heart rate
HRV: Heart rate variability
HUT: Head-up tilt
JHS: Joint hypermobility syndrome
LF: Low-frequency
LF/HF: Low-frequency/high-frequency ratio
n.u.: Normalized units
OH: Orthostatic hypotension
OI: Orthostatic intolerance
POTS: Postural orthostatic tachycardia syndrome
SBP: Systolic blood pressure
VR: Valsalva ratio.
